# De novo and rare mutations in the *HSPA1L* heat shock gene associated with inflammatory bowel disease

**DOI:** 10.1186/s13073-016-0394-9

**Published:** 2017-01-26

**Authors:** Shinichi Takahashi, Gaia Andreoletti, Rui Chen, Yoichi Munehira, Akshay Batra, Nadeem A. Afzal, R. Mark Beattie, Jonathan A. Bernstein, Sarah Ennis, Michael Snyder

**Affiliations:** 1 Department of Genetics, Stanford University School of Medicine, Stanford, CA USA; 2 Rare Disease & LCM Laboratories, R & D Division, Daiichi Sankyo Co., Ltd, Tokyo, Japan; 3 Human Genetics and Genomic Medicine, University of Southampton, Southampton General Hospital, Southampton, UK; 4 Department of Biology, Stanford University, Stanford, CA USA; 5 Oncology Laboratories, Oncology Function, Daiichi Sankyo Co., Ltd, Tokyo, Japan; 6 Department of Paediatric Gastroenterology, University Hospital Southampton NHS Foundation Trust, Southampton General Hospital, Tremona Road, Southampton, UK; 7 Department of Pediatrics, Stanford University School of Medicine, Stanford, CA USA

**Keywords:** Exome, Sequencing, Ulcerative colitis, Crohn's disease

## Abstract

**Background:**

Inflammatory bowel disease (IBD) is a chronic, relapsing inflammatory disease of the gastrointestinal tract which includes ulcerative colitis and Crohn's disease. Genetic risk factors for IBD are not well understood.

**Methods:**

We performed a family-based whole exome sequencing (WES) analysis on a core family (Family A) to identify potential causal mutations and then analyzed exome data from a Caucasian pediatric cohort (136 patients and 106 controls) to validate the presence of mutations in the candidate gene, heat shock 70 kDa protein 1-like (*HSPA1L*). Biochemical assays of the de novo and rare (minor allele frequency, MAF < 0.01) mutation variant proteins further validated the predicted deleterious effects of the identified alleles.

**Results:**

In the proband of Family A, we found a heterozygous de novo mutation (c.830C > T; p.Ser277Leu) in *HSPA1L*. Through analysis of WES data of 136 patients, we identified five additional rare *HSPA1L* mutations (p.Gly77Ser, p.Leu172del, p.Thr267Ile, p.Ala268Thr, p.Glu558Asp) in six patients. In contrast, rare *HSPA1L* mutations were not observed in controls, and were significantly enriched in patients (*P* = 0.02). Interestingly, we did not find non-synonymous rare mutations in the HSP70 isoforms *HSPA1A* and *HSPA1B*. Biochemical assays revealed that all six rare *HSPA1L* variant proteins showed decreased chaperone activity in vitro. Moreover, three variants demonstrated dominant negative effects on HSPA1L and HSPA1A protein activity.

**Conclusions:**

Our results indicate that de novo and rare mutations in *HSPA1L* are associated with IBD and provide insights into the pathogenesis of IBD, and also expand our understanding of the roles of HSP70s in human disease.

**Electronic supplementary material:**

The online version of this article (doi:10.1186/s13073-016-0394-9) contains supplementary material, which is available to authorized users.

## Background

Inflammatory bowel disease (IBD) is a complex multifactorial disease that includes ulcerative colitis (UC) and Crohn’s disease (CD). The etiology and pathogenesis of IBD are incompletely understood, and treatment of IBD can be difficult and is often unsuccessful. Although environmental factors likely play a significant role in the pathogenesis of IBD, multiple twin cohort studies [1, 2] suggest that genetic factors also contribute to IBD susceptibility. Genome-wide association studies (GWASs) have identified more than 163 risk loci for this disease [3–5] which include *NOD2*, *BACH2*, *IL23R*, *CARD9*, and human leukocyte antigen (HLA) loci. However, these common susceptibility genes together only account for 13.6% of CD and 7.5% of UC heritability [6]. One potential explanation for this heritability gap is that GWASs typically evaluate common genetic variants with minor allele frequency (MAF) > 0.05, whereas rare variants (MAF < 0.01) are often not assessed in these studies. Another likely reason for non-replication of association signals is linkage disequilibrium (LD) differences and other environmental contributions in different population groups [7].

We previously reported a wide spectrum of rare and potentially damaging variants in known IBD susceptibility genes (excluding the HLA super-locus) identified through whole exome sequencing analysis in eight patients with pediatric IBD [8]. In the present study, we report a de novo variant identified in the *HSPA1L* gene in a core family (Family A), as well as further five additional rare (MAF < 0.01), non-synonymous variants in this gene identified in a Caucasian cohort of 136 patients with pediatric IBD. In contrast, rare non-synonymous mutations were not observed in 106 controls. Moreover, we demonstrated that all six de novo and rare variants had decreased refolding activity in in vitro assays, with three of them showing dominant negative effects.

*HSPA1L*, a member of the 70-kD heat shock protein family (HSP70), is located within the HLA class III region 6p21, which has been reported as a risk locus for IBD by GWASs on Indian and Japanese populations [9, 10]. The HSP70 proteins play multiple roles in protein quality control of the cell, including refolding denatured proteins, preventing aggregation, and intracellular protein transport. In addition, HSP70s have been shown to modulate inflammatory response [11] and exert anti-apoptotic functions [12] by inhibiting apoptosis regulating proteins, both of which are closely related to IBD pathogenesis. However, the distinct role of each HSP70 family member is not well understood, and their potential role in IBD has not been established. Moreover, clinically the expression of HSP70s was reported to be unregulated in the intestine of patients with IBD [13]. Our study represents the first report on the association between IBD and de novo and rare non-synonymous mutations in the heat shock protein HSPA1L, thereby demonstrating a functional role for this protein in IBD and expanding our knowledge of the role of these proteins in human disease.

## Methods

### Cases and samples

Written informed consent was provided by an attending parent or legal guardian for pediatric participants. This study was approved by the Institutional Review Boards of Stanford University or the Southampton and South West Hampshire Research Ethics Committee (REC) (09/H0504/125) and University Hospital Southampton Foundation Trust Research & Development (RHM CHI0497).

For Family A, the proband was diagnosed with UC at the age of 16 and there was no family history of UC. A summary of the patient phenotype and characteristics is given in Table 1.

**Table 1:** Summary of patient phenotypes and characteristics with HSPA1L mutation of interest

Sample ID	HSPA1L mutation	Age at diagnosis (years)	Sex	Disease	Phenotype description	Ethnicity	Surgery	Family history
12 s^a^	p.Ser277Leu (c.830C > T)	16	Female	UC	Initially left-sided colitis and proctitis, subsequently, pancolitis	Northern, Eastern European, and Middle-Eastern mixed ancestry	-	-
PR0034^b^	p.Glu558Asp (c.1674A > T)	13	Male	CD	Nonstricturing ileocolonic	White British	+	-
PR0142^b^	p.Gly77Ser (c.229G > A)	13	Male	UC	Extensive mild to moderate pancolitis; maternal grandmother has UC	Polish	-	+
PR0151^b^	p.Ala268Thr (c.802G > A)	13	Female	CD	Panenteric colitis	White British	-	-
PR0156^b^	p.Thr267Ile (c.800C > T)	15	Male	CD	Terminal ileitis	White British	-	-
PR0161^b^	p.Ala268Thr (c.802G > A)	10	Female	UC	Extensive mild to moderate pancolitis and autoimmune sclerosing cholangitis; sister has UC (Dx age 13 years)	White British	-	+
PR0244^b^	p.Leu172del (c.515_517del)	13	Female	IBDU	Mild chronic inactive gastritis	White British	-	-

*UC* ulcerative colitis, *CD* Crohn's disease, *IBDU* inflammatory bowel disease unclassified

^a^From Family A

^b^From IBD cohort

For the Soton pediatric inflammatory bowel disease (PIBD) cohort, children diagnosed with PIBD were recruited through University Hospital Southampton (UHS). All children aged below 18 years at time of diagnosis were eligible to join the study. Diagnosis was established according to the Porto criteria, as previously described [8]. Clinical data and venous blood samples were collected. Parents and relatives diagnosed with IBD were also routinely recruited.

Detailed patient clinical phenotypes are described in Additional file 1.

### Whole exome sequencing and data analysis

For Family A, whole exome sequencing and data analysis were performed as previously described [14] with slight modifications. In brief, whole exome enrichment was performed with the Agilent SureSelect Human All Exon V5 + UTRs kit (Agilent Technologies, Santa Clara, CA, USA) and sequenced with the Illumina HiSeq 2000 sequencer (Illumina, San Diego, CA, USA). Paired-end, 101-b short reads generated from each library were mapped to the reference genome hg19 with the Burrows-Wheeler Aligner (version 0.7.7), and variants were called with the Genome Analysis Toolkit [15] (GATK; version 1.6-13-g91f02df). Called single nucleotide variants (SNVs) and Indel variants were further annotated with ANNOVAR [16] (version 2013Aug23). Potentially damaging Indels were predicted with the Scale Invariant Feature Transform (SIFT) [17] (<0.05) and PolyPhen-2 [18] (>0.85) algorithms.

For the PIBD cohort, whole exome capture was performed using the Agilent SureSelect Human All Exon 51 Mb (versions 4 and 5) capture kit. Raw data generated from paired-end sequencing protocols were aligned against hg19 using Novoalign (novoalign/2.08.02) as previously described [8, 19]. Mapping steps produced parameters for each sequenced position, such as base quality, coverage, alternative allele, reference allele, and the number of reads at that position. Sequence coverage for each sample was calculated with in-house customized scripts that applied the BEDTools [20] package (v2.13.2). Summary statistics for each individual are listed in Additional file 2. PICARD (picard/1.97) was used to remove duplicate reads and SAMtools [21] mpileup (samtools/0.1.18) was used to call single-nucleotide polymorphisms (SNPs) and short Indels from the alignment file. Variations with read depth <4 were excluded. Good-quality bases with a phred score >20 were retained for downstream analysis [22, 23]. ANNOVAR (annovar/2013Feb21) [16] was applied for variant annotation. A bespoke script was used to assign individual variants as “novel” if they were not previously reported in the dbSNP137 databases [24], 1000 Genomes Project phase one (1KG) [25], the Exome Variant Server (EVS) of European Americans of the NHLI-ESP project with 6500 exomes (http://evs.gs.washington.edu/EVS/), in 46 unrelated human subjects sequenced by Complete Genomics (46 CG) [26] or in the Southampton database of reference exomes. Resultant variant files for each subject were subjected to further in-house quality control tests to detect DNA sample contamination and ensure sex concordance by assessing autosomal and X chromosome heterozygosity. Variant sharing between all pairs of individuals was assessed to confirm that subjects were not related. Sample provenance was confirmed by application of a validated panel developed specifically for exome data [27]. Following our first process of high-quality variant detection, FASTQ raw data for the PIBD cohort were further analyzed to investigate the contribution of non-uniquely mapped reads. These reads are considered poor quality and usually discarded. However, it is possible that the analysis of these reads might impact identification of SNPs and Indels in highly homologous genes such as *HSPA1L*, *HSPA1A*, and *HSPA1B*. The raw data generated from the paired-end sequencing protocol were realigned against hg19 using Novoalign with the option to report all alignment types. PICARD was not used to remove duplicate reads, and any SNP or Indel was retained in the downstream analysis regardless of depth or phred score.

### Variants in *HSPA1L*, *HSPA1A*, and *HSPA1B*

Information for all variants called in *HSPA1L*, *HSPA1A*, and *HSPA1B* genes was collected for 136 PIBD patients and 106 controls (Additional file 3).

**Fig. 1:**
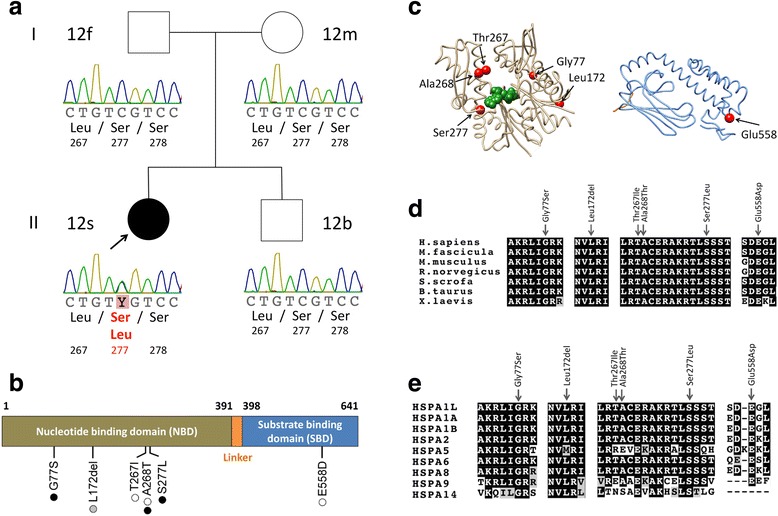
De novo and rare variants in HSPA1L. **a** The pedigree and Sanger traces of Family A. The patient with ulcerative colitis (*filled symbol*) has a de novo heterozygous mutation of c.830C > T (encoding p.Ser277Leu). **b** Schematic representation of the *HSPA1L* gene and de novo or rare variants with the number of patients identified in Family A and 136 IBD cohort. *Black*, *white*, and *gray* circles represent ulcerative colitis, Crohn's disease, and IBD unclassified, respectively. **c** The identified rare variants (*left*) on the structure of nucleotide binding domain (*NBD*) of HSPA1L (PDB entry codes: 3GDQ [45]) and (*right*) on homology-based model of substrate binding domain (*SBD*) of HSPA1L created by using Phyre2 [46]. The variant sites are shown in *red*, and ADP and PO_4_ are depicted as a space-filling representation in *green*. **d** Amino acid conservation of HSPA1L among species. **e** Amino acid conservation among paralogs of HSPA1L in human. Amino acid sequences were aligned using Clustal Omega and annotated using BOXSHADE (**d**, **e**)

Rare (MAF <0.01), non-synonymous *HSPA1L* mutations were selected and verified by Sanger sequencing in the proband and relatives where applicable (Fig. 1a, Additional file 4).

### Burden of mutation testing across HSPA genes

Whole exome sequencing data were available on 146 children diagnosed with IBD. The Soton analytical group also has access to germline exome sequence data for 126 unrelated patients with no inflammatory-related disease. In order to minimize bias for association analysis, we conducted multidimensional scaling (MDS) analysis in PLINK (plink/1.07) [28] on a combined set of patients and controls and excluded non-Caucasian samples (Additional file 5). This reduced the number of cases to 136 and controls to 106. All variants identified in any individual for *HSPA1L*, *HSPA1A*, and *HSPA1B* genes were positively called in all samples across the PIBD patients and controls, and these genotypes were selected for further analysis.

To detect association between genetic variant and disease status, a gene-based test (the sequence kernel association optimal unified test, SKAT-O [29]) was performed. SKAT-O is used for the joint assessment of the contribution of rare and common variations within a genomic locus with a trait [29]. Specifically, SKAT-O encompasses both a burden test and a SKAT [29] test to offer a powerful way of conducting association analysis on combined rare and common variations, as single variant tests are often underpowered due to the large sample size needed to detect a significant association.

In order to run the test, genotype information (homozygous alternative, homozygous reference, or heterozygous status) was retrieved using customized scripts applying SAMtools [21], VCFtools [30], and BEDTools [20] packages. All variant sites across the coding regions of *HSPA1L*, *HSPA1A*, and *HSPA1B* genes were used to generate a VCF file for each of the 136 cases and 106 unrelated, germline controls.

Variants were excluded using VCFtools [30] if they deviated significantly from Hardy-Weinberg equilibrium status (*P* < 0.001) in the control group. VCF files containing genotype information for all cases and controls were merged and annotated.

To conduct the test, a group file of non-synonymous and in-frame deletion only variants was created for each of the three genes. SKAT-O was conducted excluding synonymous variants, as these are less likely to impact the protein function, as previously described [31–33]. SKAT-O was executed with the small sample adjustment, by applying a MAF threshold of 0.01 to define rare variations within the whole cohort, and using default weights. The Efficient and Parallelizable Association Container Toolbox for Sequence Data (EPACTS) software package [34] was used to perform this test.

### Expression of the HSPA1L protein

The *HSPA1L* gene consists of a single exon. The coding region was amplified using genomic DNA from the affected patient 12 s of Family A by PCR, and the PCR products were cloned into a pCR-Blunt II-TOPO vector (Invitrogen). After cloning, a common single nucleotide variant rs2227956 was reverted to its reference sequence (wild type, WT) by using a QuikChange II Site-Directed Mutagenesis Kit (Agilent Technologies, La Jolla, CA, USA), and p.Lys73Ser (c.218A > G, c.219A > C), p.Gly77Ser (c.229G > A), p.Leu172del (c.515-517del), p.Thr267Ile (c.800C > T), p.Ala268Thr (c.802G > A), p.Ser277Leu (c.830C > T), and p.Glu558Asp (c.1674A > T) mutants were generated and subsequently cloned into the pGEX-6P-1 vector (GE Healthcare, Waukesha, WI, USA) at the *Bam*HI-*Not*I restriction site. All sequences were confirmed by Sanger sequencing analysis at the Protein and Nucleic Acid Facility (Stanford University).

The resulting vector was transformed into *Escherichia coli* strain BL21 (New England Biolabs, Ipswich, MA, USA), and recombinant fusion protein with a glutathione S-transferase (GST) tag was expressed by induction with 0.1 mM of isopropyl-β-thiogalacto-pyranoside (Sigma) for 5–6 hours at 28 °C. Cells were pelleted and resuspended in lysis buffer (50 mM pH7.5 Tris–HCl, 150 mM NaCl, 0.05% NP-40) and lysed with 0.25 mg/mL lysozyme (EMD Millipore, Billerica, MA, USA) on ice for 30 minutes. The samples were then sonicated and centrifuged at 20,000 × g for 20 minutes. The resulting supernatants were incubated with Glutathione Sepharose 4B beads (GE Healthcare) for 3 hours at 4 °C. Recombinant protein-bound beads were subsequently washed with lysis buffer and incubated with PreScission Protease (GE Healthcare) overnight at 4 °C. Protein concentration was measured by Bradford assay. The eluted protein was concentrated as necessary by using Amicon Ultracel-3 K columns (Millipore). The purified protein samples were aliquoted and stored at −80 °C.

### In vitro chaperone assay

In vitro chaperone activity was measured with the HSP70/HSP40 Glow-Fold Protein Refolding Kits (K-290, Boston Biochem, Cambridge, MA, USA) according to the manufacturer’s protocol with modifications. In brief, recombinant HSPA1L protein (4 μM), a 1:1 mixture of recombinant HSPA1L WT protein (2 μM) and HSPA1L mutant protein, or a 1:1 mixture of HSPA1A protein (2 μM, Boston Biochem) and recombinant HSPA1L protein (2 μM) was used to test for refolding efficiency of heat-denatured Glow-Fold Substrate protein. Luminescence measurements were taken using a TECAN infinite 200 microplate reader (TECAN Austria GmbH, Salzburg, Austria) at indicated time points within 1 minute of mixing with luciferin reagent. Refolding activity was calculated by subtracting the luminescence at time 0 (before refolding reaction) from that at 120 minutes (after refolding reaction). The refolding activity of each control at 120 minutes was set as 100%. Data were compared between the control and test samples using Dunnett's multiple comparison test.

## Results

### Family-based whole exome sequencing analysis revealed a de novo mutation in *HSPA1L*

We analyzed the exomes of Family A, comprising the affected proband (12 s) diagnosed with UC, both unaffected parents, and an unaffected sibling. After excluding implausible genes such as those encoding olfactory receptors and mucins, and applying the *in silico* prediction algorithms (SIFT < 0.05 and PolyPhen-2 > 0.85), we found a single de novo heterozygous mutation, c.830C > T (encoding p.Ser277Leu) affecting the gene *HSPA1L* only in 12 s but not in the other family members (Fig. 1a, Table 1, Additional file 6). All genotypes were confirmed by Sanger sequencing (Fig. 1a). The mutation resides at a nucleotide binding site (Fig. 1b and c) and is highly conserved between species (Fig. 1d) and within paralogous members of the human HSP70 family (Fig. 1e). Other candidate genes with homozygous or compound heterozygous deleterious mutations were not evident from the SNV and Indel variant calls (Additional file 6), thereby making *HSPA1L* the lead candidate gene for the IBD phenotype.

### Confirmation of additional rare mutations in *HSPA1L* in larger cohort of patients with IBD

To determine the prevalence of rare *HSPA1L* mutations in patients with IBD, we analyzed the exomes of an additional 136 IBD patients and 106 exomes of non-IBD control subjects. We identified 14 *HSPA1L* variants across the exomes of children diagnosed with IBD and controls (Table 2). Four were rare (MAF <0.01) non-synonymous mutations and one was a novel in-frame 3-base pair (bp) deletion, which were found only in IBD patients. Of the remaining nine variants, two were low frequency (MAF 0.01–0.05) non-synonymous mutations, two were common (MAF >0.05) non-synonymous variants, and five were synonymous. Thus, the rare (MAF <0.01, p.Glu558Asp, p.Gly77Ser, p.Ala268Thr, p.Thr267Ile) and novel(p.Leu172del) non-synonymous variants were observed in cases only.

**Table 2:** Variants found in patients with IBD and controls in *HSPA1L* (no filtering applied)

Base pair location in hg19	Variant type	Nucleotide change	Protein change	phylop	1-sift	PolyPhen2	Grantham score	dbSNP137	Frequency in 1KG Project	Cases^a^ genotypes (homozygous reference allele, heterozygous, homozygous alternative allele)	Controls^b^ genotypes (homozygous reference allele, heterozygous, homozygous alternative allele)	MAF within combined cases and controls cohort
31779233	ifd	c.515_517del	p.Leu172del	.	.	.	.	.	.	135,1,0	106,0,0	0.0020^c^
31778076	ns	c.1674A > T	p.Glu558Asp	0.108385	T	B	C	.	0.0000089^e^	135,1,0	106,0,0	0.0020^c^
31779521	ns	c.229G > A	p.Gly77Ser	0.936178	D	D	MC	rs368138379	0.0000770^d^	135,1,0	106,0,0	0.0020^c^
31778948	ns	c.802G > A	p.Ala268Thr	0.997482	D	D	MC	rs34620296	0.0014000	134,2,0	106,0,0	0.0041^c^
31778950	ns	c.800C > T	p.Thr267Ile	0.998993	D	D	MC	rs139868987	0.0014000	135,1,0	106,0,0	0.0020^c^
31779728	ns	c.22G > C	p.Ala8Pro	0.995889	D	D	C	rs9469057	0.0130000	136,0,0	103,3,0	0.0061
31778077	ns	c.1673A > C	p.Glu558Ala	0.995982	T	P	MR	rs2227955	0.0480000	129,7,0	98,8,0	0.0309
31777946	ns	c.1804G > A	p.Glu602Lys	0.997651	D	B	MC	rs2075800	0.2900000	57,57,22	48,49,9	0.3471
31778272	ns	c.1478C > T	p.Thr493Met	0.008994	T	B	MC	rs2227956	0.8800000	6,33,97	2,23,81	0.1487
31778697	sn	c.1053G > C	p.Leu351Leu	.	.	.	.	rs199780750	0.0000400^e^	135,1,0	106,0,0	0.0020
31779003	sn	c.747G > A	p.Arg249Arg	.	.	.	.	rs116768554	0.0027000	135,1,0	106,0,0	0.0020
31778322	sn	c.1428C > T	p.Ile476Ile	.	.	.	.	rs35347921	0.0040000	135,1,0	106,0,0	0.0020
31778831	sn	c.919 T > C	p.Leu307Leu	.	.	.	.	rs35326839	0.0200000	133,3,0	102,4,0	0.0144
31778529	sn	c.1221G > A	p.Thr407Thr	.	.	.	.	rs2075799	0.1400000	123,13,0	90,14,2	0.0640

14 variants ordered by variant type and within type ordered by frequency in 1000 Genome Project

^a^Soton PIBD exomes, *n* = 136

^b^Soton controls, *n* = 106

^c^Variants used in the SKAT-O test

^d^Frequency in NHLBI ESP

^e^Frequency in ExAC Browser

Dots denote missing data

*ns* non-synonymous, *sn* synonymous, *ifd* in-frame deletion

*B* benign, *C* conservative, *D* deleterious, *MC* moderately conservative, *MR* moderately radical, *P* possibly damaging, *T* tolerated

Of interest, the four rare non-synonymous mutations (p.Gly77Ser, p.Thr267Ile, p.Ala268Thr, and p.Glu558Asp) and the novel in-frame deletion (p.Leu172del) reside at highly conserved residues throughout speciation and human paralogs (Fig. 1d and e). The p.Gly77Ser and p.Leu172del variants are adjacent to the nucleotide binding site and in the beta sheet structure (http://www.uniprot.org/uniprot/P34931) respectively; p.Thr267Ile and p.Ala268Thr are located at a nucleotide exchange factor binding domain, and p.Glu558Asp resides in a substrate binding domain. These five variants were deemed to be of highest functional interest and were verified by Sanger sequencing in the probands and all relatives for whom DNA was available (Additional file 4). The *HSPA1L* mutation p.Ala268Thr was also confirmed in the patient’s affected sister, who is also diagnosed with UC.

Together with the index family case, these results indicate that five of six *HSPA1L* IBD mutations may affect nucleotide binding or exchange.

### Mutations in *HSPA1A* and *HSPA1B*

We also examined the highly homologous *HSPA1A* and *HSPA1B* genes in the exome sequenced cohort. Although *HSPA1L* is expressed at a low level in the intestine, *HSPA1A* and *HSPA1B* are abundantly expressed in this tissue. We found two common synonymous variants in *HSPA1A* and five synonymous variants in *HSPA1B*, of which three were at low frequency (MAF 0.01–0.05) in the 1000 Genome Project (Additional file 7).

We also performed variant calling only on the reads that are non-uniquely mapped to the *HSPA1A* and *HSPA1B* as described in Methods. Nevertheless, we did not find non-synonymous mutations in either of the *HSPA1A* and *HSPA1B* homologs.

### Joint rare variant association test

We conducted a gene-based test for assessing the combined association of coding novel, rare, and low frequency mutations between affected and unaffected individuals within the whole cohort. This analysis was limited to variants most likely to impact protein function and discounted synonymous changes. For *HSPA1L*, SKAT-O testing was conducted on the four rare non-synonymous mutations (p.Gly77Ser, p.Thr267Ile, p.Ala268Thr, and p.Glu558Asp) and one novel in-frame deletion (p.Leu172del) (see Methods). The test showed a significant association between *HSPA1L* variants and the IBD phenotype (*P* = 0.024, Additional file 8). When the SKAT-O test was repeated to include the two low frequency non-synonymous mutations (p.Ala8Pro and p.Glu558Ala) in addition to the five rare mutations, the association remained significant (*P* = 0.034, Additional file 9). Since we did not observe any non-synonymous variants in *HSPA1A* and *HSPA1B*, we did not conduct the SKAT-O test for these genes. Overall, these analyses indicate that the rare mutations in *HSPA1L* are associated with IBD. The fact that the majority of mutations reside in specific domains (i.e., at nucleotide binding or exchange) further suggests that these variants are not randomly associated with IBD and likely to be causative mutations.

### In vitro chaperone activity assays showed defective protein function

**Fig. 2:**
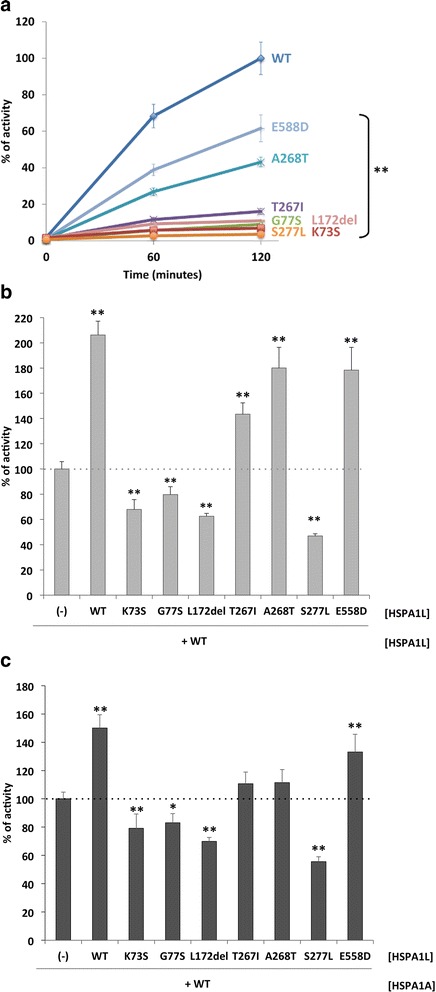
Effects of the HSPA1L variants on HSP70/HSP40-mediated refolding heat-denatured luciferase. **a** Reactivation of heat-denatured luciferase in the presence of each HSPA1L variant (4 μM). Luciferase activity in the presence of HSPA1L WT at 120 minutes was set as 100%. ** indicates *P* < 0.01 for the comparison between HSPA1L WT and each variant by Dunnett’s test (*n* = 3–6). **b** Dominant negative effects of Gly77Ser, Leu172del, and Ser277Leu in refolding activity of each HSPA1L variant (2 μM) in the presence of HSPA1L WT (2 μM). Refolding activity of HSPA1L WT (2 μM) only was set as 100%. ** indicates P < 0.01 for the comparison between HSPA1L WT only and each variant by Dunnett’s test (*n* = 3–6). **c** Dominant negative effects of Gly77Ser, Leu172del, and Ser277Leu in refolding activity of each HSPA1L variant (2 μM) in the presence of HSPA1A WT (2 μM). Refolding activity of HSPA1A WT (2 μM) only was set as 100%. * or ** indicates *P* < 0.05 or *P* < 0.01, respectively, for the comparison between HSPA1A WT only and each variant by Dunnett’s test (*n* = 3–4). The activity of the previously known mutation Lys73Ser was measured as a positive control for loss-of-function and dominant negative mutant. The bars represent the standard deviation. Data are representative of two independent experiments

In order to evaluate the effects of the rare non-synonymous variants on chaperone function, we measured the refolding of heat-inactivated luciferase substrate using recombinant HSPA1L proteins. In vitro functional analyses revealed that all six variants resulted in partial or complete loss of in vitro HSPA1L chaperone activity compared with the WT control (Fig. 2a). Among them, three variants (p.Gly77Ser, p.Leu172del, and p.Ser277Leu) showed complete loss of activity, of which two are located at or near the nucleotide binding site.

The HSPA1L mutations were heterozygous in each patient. To evaluate whether the mutant alleles have dominant negative effects on WT HSPA1L protein, we compared the activity of a 1:1 mixture (molar) of WT and mutant protein with the activity of HSPA1L WT alone. We also measured the activity of the previously known loss-of-function mutation p.Lys73Ser (equivalent to p.Lys71Ser in HSPA1A [35]) as a control for the dominant negative effect. As shown in Fig. 2b, the identified variants (p.Gly77Ser, p.Leu172del, and p.Ser277Leu) each exhibited dominant negative effects as did the positive control (p.Lys73Ser), whereas p.Thr267Ile, p.Ala268Thr, and p.Glu558Asp showed additive effects (i.e., reduced activity). In addition, the three variants (p.Gly77Ser, p.Leu172del, and p.Ser277Leu) also dominant negatively suppress the activity of WT HSPA1A in a 1:1 mixture (molar) of WT HSPA1A and mutant HSPA1L protein (Fig. 2c), suggesting that these variants may affect the activity of other HSP70 chaperone family proteins.

## Discussion

In this exome sequencing study, two phases of analysis were conducted. The first phase was a family-based analysis, which revealed a de novo, novel *HSPA1L* mutation as a candidate potential causative mutation in the index IBD patient with no family history of IBD. The second phase was the validation analysis of rare *HSPA1L* mutations using a moderate number of exomes from cases and controls. In the validation study, five different novel or rare non-synonymous variants were identified in 6 out of 136 patients with IBD, whereas no rare variants were found in 106 controls. The large number of rare variants observed in *HSPA1L* may also result from selective pressure at the HSPA1L locus, which is supported by the gene-environment interaction model underlying IBD. The rare *HSPA1L* mutations observed in the Southampton cohort were inherited from unaffected parents, which might indicate that a potential cumulative effect from other genetic defects may act either independently or together with HSPA1L to influence disease susceptibility.

Although the minor allele frequency for each of these five variants is too low to assess its association to the disease individually, a gene-based SKAT-O test revealed a significant burden of mutation (*P* = 0.024) when assessing non-silent rare variants observed in *HSPA1L*. The association maintained significance when reassessed to include all rare and low frequency variants (*P* = 0.034).

The *HSPA1L* gene is located in the MHC class III region, which is within the IBD3 locus, a known genetic linkage region for both UC and CD [36]. Likewise, in our study, rare *HSPA1L* mutations were observed both in UC, CD, and IBDU patients (Fig. 1b). These data suggest that HSPA1L might play a common pathogenic role in IBD. HSPA1L is constitutively expressed, but its expression is at a lower level compared to other members of the HSP70 family, such as HSPA1A and HSPA1B [37]. Although the distinctive role of each isoform and the substrate protein specificity for each HSP70 family member have not been well studied, it is reported that each HSP70 has binding preferences to purified peptides [38]. Also in a recent study, Hasson et al. demonstrated that HSPA1L, and not HSPA1A, promotes translocation of parkin to damaged mitochondria [39], which is required for mitophagy, suggesting that HSPA1L has specific protein substrates and functions. Further analysis of IBD phenotype, response to therapy, and histopathological data of patients with or without HSPA1L mutation may lead to a better understanding of disease mechanisms.

Recently, homodimerization of HSP70 (or DnaK in E coli) [40, 41] and its relevance with the protein function [42] have been reported. One possible explanation of IBD pathogenesis is that mutated HSPA1L protein perturbs the HSP70 chaperone system dominant negatively by preventing the dimerization [40, 42] and blocks its protective effects to stress in the colon, which results in the loss of normal intestinal barrier function against invasive bacteria or bacterial toxins [43]. In mice, Hspa1a and Hspa1b double knockout mice were phenotypically normal; however, when treated with dextran sulfate sodium and exposed to oxidative stress, they exhibited colitis [44]. Further studies using mice with the specific *HSPA1L* mutations identified in patients with IBD will improve our understanding of the pathogenesis of IBD.

Among the identified variants, p.Gly77Ser, p.Leu172del, and p.Ser277Leu were more deleterious in that they showed almost complete loss of function and significant dominant negative effects in in vitro assays. This severity is consistent with their low allele frequency (i.e., de novo or novel) in the human population (Table 2). We hypothesized that these deleterious variants might be associated with more severe clinical observations, such as very early onset of IBD or severe symptoms; however, no such correlation was evident in our modest-sized group of subjects. For example, patient 12 s with a severe p.Ser277Leu mutation had relatively late onset at age 16, whereas patient PR0161, who had a less-deleterious p.Ala268Thr mutation, was diagnosed at age 10 years. We speculate that other genetic and/or environmental factors are likely to contribute to disease severity.

It is remarkable to note that, unlike *HSPA1L*, non-synonymous variants were not found in the *HSPA1A* or *HSPA1B* genes (Additional file 7) given that HSP70 family proteins are highly homologous in sequence. This was assessed in two ways: First we mapped short reads using conventional alignment tools as described in Methods; however, we were unable to completely exclude technical limitation in mapping reads to highly homologous regions to detect variation within these genes. Thus, we also processed the unmapped reads on the genes and searched the rare non-synonymous mutations extensively (see Methods). With this additional effort, we could not find any rare non-synonymous mutations in *HSPA1A* and *HSPA1B*, indicating that the mutations in HSP70 are *HSPA1L*-specific. This result is important both in further understanding of the pathogenesis of IBD and in developing drug strategy for patients with IBD who harbor the HSPA1L mutations.

Through whole exome sequencing analysis of Family A and Caucasian IBD cohorts, we found a statistically significant association between IBD and rare mutations in the *HSPA1L* gene. These variants caused loss of function of the HSPA1L protein to varying degrees, and three of them also exhibited dominant negative effects on the wild-type protein, which may in turn contribute to the disease phenotype. The frequency of HSPA1L mutations in our cohort (4.4%) is high enough that they could potentially be used for the purpose of genetic risk assessment. In addition, we believe that these findings may provide insights into the pathogenesis and treatment of IBD as well as the general role of HSP70 proteins in human biology and disease. They can also suggest new directions for the development of therapeutics by inactivating *HSPA1L* activity in patients with a dominant negative mutation.

## Conclusions

Our results indicate that *de novo* and rare mutations in HSPA1L are associated with IBD. These findings provide insights into the pathogenesis and treatment of IBD, as well as expand our understanding of the roles of HSP70s in human disease.

## Additional files

Additional file 1: Patient profiles. (DOCX 132 kb)
Additional file 2: Summary statistics for exome sequencing: mapping and coverage. (DOCX 90 kb)
Additional file 3: Percentage of bases covered for *HSPA1L*, *HSPA1A*, and *HSPA1B* in the Agilent SureSelect V4 and V5 kits. (DOCX 42 kb)
Additional file 4: Sanger traces of each of the four variants of interest found across six pedigrees. (DOCX 1252 kb)
Additional file 5: Multidimensional scaling (MDS) across five ethnic groups from 1000 Genome Project, 146 pediatric IBD cases, and 126 non-IBD controls. (DOCX 261 kb)
Additional file 6: Homozygous and heterozygous mutations unique to the index patient with ulcerative colitis (12 s). (DOCX 126 kb)
Additional file 7:
*HSPA1A* and *HSPA1B* variants identified in patients wth IBD and controls (no filtering applied). (DOCX 68 kb)
Additional file 8: Results of SKAT-O within HSPA1L using non-synonymous and non-frameshift rare (MAF < 0.01) variants. (DOCX 49 kb)
Additional file 9: Results of SKAT-O within HSPA1L using non-synonymous and non-frameshift variants, excluding common variants (MAF > 0.05). (DOCX 51 kb)

## Abbreviations

CD: Crohn’s disease
GWAS: Genome-wide association study
HSP70: 70 kDa heat shock protein
HSPA1L: Heat shock 70 kDa protein 1-like
IBD: Inflammatory bowel disease
IBDU: Inflammatory bowel disease unclassified
MAF: Minor allele frequency
SKAT-O test: Sequence kernel association optimal unified test
UC: Ulcerative colitis
WES: Whole exome sequencing
