# Supplementary material for: De novo and rare mutations in the HSPA1L heat shock gene associated with inflammatory bowel disease
Source: Genome Med. 2017 Jan 26;9:8. doi: 10.1186/s13073-016-0394-9 (PMC5270254; doi:10.1186/s13073-016-0394-9)
Supplement: Additional file 1: — Patient profiles. (DOCX 132 kb) [file 13073_2016_394_MOESM1_ESM.docx]

**Additional file 1**

**Patient Profiles**

12s in Family A was identified with the p.Ser277Leu *de novo* mutation in *HSPA1L*. She was diagnosed with UC at age 16. She was initially treated with oral and rectal 5-aminosalicylic acid and subsequently has been on 5-aminosalicylic acid and azathioprine. There is no family history of IBD. She carries a rare (MAF = 0.0016) mutation (p.Leu358Trp) in the *NKX2-3* gene, a member of the *NKX* family of homeodomain-containing transcription factor, which is known as a IBD susceptibility gene by GWAS studies. She also harbors a novel non-synonymous (p.Pro466Ser) mutation in the *TXK* gene that is also known as a susceptibility gene for IBD and Behcet's disease and a non-synonymous mutation within *IKBKG* (p.Glu57Lys) which is a gene involved in the NOD2 pathway.

PR0034, harboring the *HSPA1L* p.Glu558Asp, was diagnosed with Crohn’s disease at age 13 years. He presented with diarrhea, abdominal pain and a significant weight loss. Initial endoscopy and radiological investigations showed ileo-colonic Crohn’s disease. He was initially started on enteral nutrition, but responded poorly necessitating treatment with intravenous steroids. Due to poor nutritional improvement, he needed a prolonged support with naso-gastric tube feeds. He was subsequently started on thiopurines following which he remained stable for a few months, but then developed significant relapses necessitating treatment with biologics. Further endoscopic and radiological investigations revealed structuring disease for which, he underwent a de-functioning ileostomy and right hemicolectomy due to failure of medical management at age 13. He is currently stable and in remission on infliximab and azathioprine.

PR0142, a male patient diagnosed with ulcerative colitis at 13 years of age. He presented with bloody diarrhea for over a year. Initial endoscopy showed moderate degree of pan-colitis with backwash ileitis. He was initially treated with steroids and then subsequently maintained in remission on 6-mercapturine. There was a family history of colitis in the maternal grand-mother. He carries the *HSPA1L* p.Gly77Ser mutation and two damaging heterozygous non-synonymous mutations in *DOK3* - a gene involved in the B-cell receptor signaling pathway. However, discontinuous exome sequencing data is incapable of resolving phase and we cannot confirm compound heterozygosity without additional sequencing. This patient also carries a potential damaging variant in *CARD11* (p.Arg974Cys) and in *LRBA* (p.Arg1997Cys). *CARD11* is a UC associated gene encoding for cytoplasmic proteins involved in the apoptotic signaling cascade and activation of NF-κI [1], while *LRBA* is involved in regulating endosomal trafficking [2].

PR0151, a female patient having the p.Ala268Thr mutation, presented at 13 years of age, with a two year history of abdominal pain and diarrhea following an infection with cryptosporidium. Endoscopy at diagnosis showed moderately severe Crohn’s disease with granulomatous gastritis, ileitis and colitis. She also had significant weight loss and a reduced height velocity at diagnosis. Following initial treatment with Modulen, then 5-azathioprine, she was subsequently started on infliximab due to recurrent disease. She has maintained remission for the last three years on a combination of azathioprine and infliximab. As phase and parental origins cannot be resolved by WES, she harbors potential compound heterozygous (p.Thr347Met and p.Leu669Gln) variants in the *ERAP2* gene that are likely to impact the antigen presentation function of the protein*.* Both variants occur at a 2% allele frequency in 1000 Genomes Project. This patient also harbors a rare possibly damaging mutation in *NDUFAF1* (p.Arg213His) that encodes a protein involved in the mitochondrial respiratory chain, and a low frequency variant in *NLRP2* (p.Ala801Thr), an inhibitor of the NF-κB signaling pathway [3].

PR0156 was diagnosed with ileal-caecal CD aged 15 years. He presented with long-standing history of abdominal pain and weight loss over several months. There was also a background of asthma and hay fever. Initial therapy with enteral nutrition and then subsequently steroids did not show significant improvement. He was started on infliximab within the first year of diagnosis due to a protracted disease course. He has maintained remission on infliximab for the last three years. He carries the *HSPA1L* p.Ala267Thr and the *NOD2* p.Arg702Trp variants in heterozygous state, the latter representing one of the three biomarkers for IBD that is associated with a twofold increase in odds ratio of CD [4]. In addition he also has a novel mutation within *FCGR2A* (p.Val253Ala) reported as possibly damaging by PolyPhen2 but as tolerated and moderate conserved by 1-SIFT and Grantham respectively. He carries two heterozygous mutations in *SLAMF1*, regulator of the microbicidal mechanisms in macrophages [5], of which variant (p.Pro333Thr) is annotated as deleterious but the second novel (p.Thr235Ala) variant is annotated as benign, and two damaging heterozygous non-synonymous mutations in *TTC7A* - a gene involved in intestinal development [6].

PR0161 is an early onset UC patient diagnosed at age 10 years with concurrent autoimmune sclerosing cholangitis. She presented with abdominal pain, rectal bleeding and deranged liver function tests. Her disease course has remained relatively stable on a combination of azathioprine, ursodeoxycholic acid and a low dose prednisolone (for the liver disease). She carries a rare *NOD2* variation (p.Arg703Cys, MAF = 0.002) located in the leucine rich domain that is implicated in intracellular receptor function for components of microbial pathogens [7]. The patient also harbors a rare frameshift insertion in *CXCL6*. This variant is not reported in public repositories (1KG, dbSNP, EVS, 46 CG [8]) but it has been observed in one other Southampton IBD pediatric proband as well as four controls (patients diagnosed with non-autoimmune conditions) within the Southampton control group of reference exomes. The *HSPA1L* mutation carried by this patient (p.Ala268Thr) was also confirmed in her affected sister who is also diagnosed with UC.

PR0244 was diagnosed with inflammatory disease unclassified (IBDU) aged 13 years. She was started on 5-azathioprine, budesonide and prednisolone. Non-resolving symptoms have necessitated continuous steroidal therapy since January 2015. She carries the *HSPA1L* p.Leu172del and a novel mutation (p.Val94Met) in the *IRF1* gene shown to regulate apoptosis, DNA damage and tumor suppression [9]. She also harbors a rare (MAF = 0.0032) non-synonymous (p.Arg313Pro) damaging variant in the *ITLN1* gene implicated in pathogen recognition [10]

1. Van Limbergen J, Radford-Smith G, Satsangi J. Advances in IBD genetics. Nat. Rev. Gastroenterol. Hepatol. 2014;11:372–85.

2. Alangari A, Alsultan A, Adly N, Massaad MJ, Kiani IS, Aljebreen A, et al. LPS-responsive beige-like anchor (LRBA) gene mutation in a family with inflammatory bowel disease and combined immunodeficiency. J. Allergy Clin. Immunol. 2012;130:481–8.e2.

3. Fontalba A, Gutierrez O, Fernandez-Luna JL. NLRP2, an Inhibitor of the NF- B Pathway, Is Transcriptionally Activated by NF- B and Exhibits a Nonfunctional Allelic Variant. J. Immunol. American Association of Immunologists; 2007;179:8519–24.

4. Lesage S, Zouali H, Cézard JP, Colombel JF, Belaiche J, Almer S, et al. CARD15/NOD2 mutational analysis and genotype-phenotype correlation in 612 patients with inflammatory bowel disease. Am J Hum Genet. 2002;70:845–57.

5. van Driel B, Liao G, Romero X, O’Keeffe MS, Wang G, Faubion WA, et al. Signaling lymphocyte activation molecule regulates development of colitis in mice. Gastroenterology. 2012;143:1544–54.e7.

6. Chen R, Giliani S, Lanzi G, Mias GI, Lonardi S, Dobbs K, et al. Whole-exome sequencing identifies tetratricopeptide repeat domain 7A (TTC7A) mutations for combined immunodeficiency with intestinal atresias. J. Allergy Clin. Immunol. 2013;132:656–64.

7. Rivas MA, Beaudoin M, Gardet A, Stevens C, Sharma Y, Zhang CK, et al. Deep resequencing of GWAS loci identifies independent rare variants associated with inflammatory bowel disease. Nat. Genet. 2011;43:1066–73.

8. Drmanac R, Sparks AB, Callow MJ, Halpern AL, Burns NL, Kermani BG, et al. Human Genome Sequencing Using Unchained Base Reads on Self-Assembling DNA Nanoarrays. Science (80-. ). 2009;327:1–7.

9. Tang R, Yang G, Zhang S, Wu C, Chen M. Opposite Effects of Interferon Regulatory Factor 1 and Osteopontin on the Apoptosis of Epithelial Cells Induced by TNF-α in Inflammatory Bowel Disease. Inflamm. Bowel Dis. 2014;20:1950–61.

10. Brown EM, Sadarangani M, Finlay BB. The role of the immune system in governing host-microbe interactions in the intestine. Nat. Immunol. 2013;14:660–7.
