# Supplementary material for: De novo and rare mutations in the HSPA1L heat shock gene associated with inflammatory bowel disease
Source: Genome Med. 2017 Jan 26;9:8. doi: 10.1186/s13073-016-0394-9 (PMC5270254; doi:10.1186/s13073-016-0394-9)
Supplement: Additional file 2: — Summary statistics for exome sequencing: mapping and coverage. (DOCX 90 kb) [file 13073_2016_394_MOESM2_ESM.docx]

**Additional file 2.** Summary statistics for exome sequencing - mapping and coverage

| Sample ID | Agilent Exome capture | Number of sequenced reads | Total no. aligned reads | Total no. unique align. | Mapped to target reads +/-150bp (%) | Mapped to target reads (%) | Target bases with coverage >1 (%) | Target bases with coverage >5 (%) | Target bases with coverage >10 (%) | Target bases with coverage >20 (%) | Mean coverage |
| --- | --- | --- | --- | --- | --- | --- | --- | --- | --- | --- | --- |
| 12s | V5+ UTRs | 87387846 | 86684829 | 66127387 | 75.93 | 70.16 | 99.8 | 99.2 | 97.7 | 92.1 | 65.14 |
| PR0034 | V5 | 44626172 | 44309916 | 43565707 | 88.16 | 75.89 | 99.28 | 98.35 | 95.87 | 86.56 | 55.43 |
| PR0151 | V4 | 36355666 | 36122486 | 35800555 | 88.72 | 83.31 | 99.85 | 99.16 | 97.39 | 91.51 | 97.21 |
| PR0161 | V4 | 50590970 | 49467147 | 49007862 | 84.3 | 76.74 | 99.68 | 98.28 | 94.77 | 83.67 | 62.04 |
| PR0142 | V4 | 67676356 | 66652672 | 66223398 | 94.55 | 85.01 | 99.83 | 99.14 | 97.57 | 92.08 | 86.13 |
| PR0156 | V5 | 46590950 | 46257701 | 45479121 | 87.42 | 75.31 | 99.26 | 98.37 | 96.08 | 87.26 | 57.04 |
| PR0244 | V5 | 48919246 | 48579280 | 47801772 | 82.28 | 99.31 | 99.27 | 98.46 | 97.21 | 91.31 | 58.94 |

Number of sequenced reads - total number of reads sequenced; Total no. aligned reads - the total number of reads aligned to the reference sequence; Total no. unique align- the number of reads that uniquely mapped to the reference sequence; Mapped to target reads +/-150bp (%)- the percentage of reads mapped ±150 base pair to the target; Mapped to target reads (%)- the percentage of reads mapped to the target sequence; Target bases with coverage >1,5,10,20- the percentage of targets with 1, 5, 10 and 20 read depth; Mean coverage - the mean of the depth coverage.
