# Supplementary material for: De novo and rare mutations in the HSPA1L heat shock gene associated with inflammatory bowel disease
Source: Genome Med. 2017 Jan 26;9:8. doi: 10.1186/s13073-016-0394-9 (PMC5270254; doi:10.1186/s13073-016-0394-9)
Supplement: Additional file 3: — Percentage of bases covered for HSPA1L, HSPA1A, and HSPA1B in the Agilent SureSelect V4 and V5 kits. (DOCX 42 kb) [file 13073_2016_394_MOESM3_ESM.docx]

**Additional file 3.** Percentage of bases covered for *HSPA1L*, *HSPA1A* and *HSPA1B* in the Agilent SureSelect V4 and V5 kits.

| Gene Name | Gene size (bp) | Coding size (bp) | Agilent V4 % gene coverage  (87 IBD cases and 56 controls) | Agilent V5 % gene coverage  (59 IBD cases and 70 controls) |
| --- | --- | --- | --- | --- |
| HSPA1L | 6,042 | 1,923 | 16.7 | 18.7 |
| HSPA1A | 2,433 | 1,923 | 5.9 | 20.0 |
| HSPA1B | 2,520 | 1,923 | 3.2 | 20.0 |
