# Supplementary material for: De novo and rare mutations in the HSPA1L heat shock gene associated with inflammatory bowel disease
Source: Genome Med. 2017 Jan 26;9:8. doi: 10.1186/s13073-016-0394-9 (PMC5270254; doi:10.1186/s13073-016-0394-9)
Supplement: Additional file 4: — Sanger traces of each of the four variants of interest found across six pedigrees. (DOCX 1252 kb) [file 13073_2016_394_MOESM4_ESM.docx]

**Additional file 4**

**Figure S4A-F.** Sanger traces of each of the four variants of interest found across six pedigrees. Genotypic state was confirmed in all available family members.

**Figure S4A. Sanger trace for Pedigree 34**


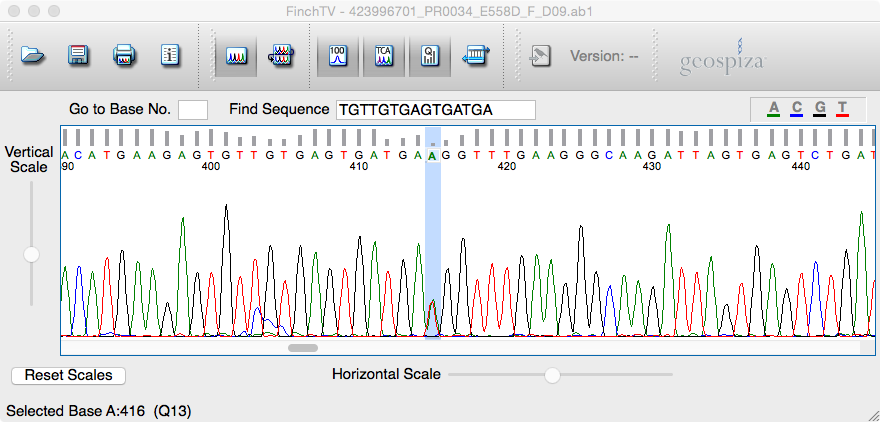

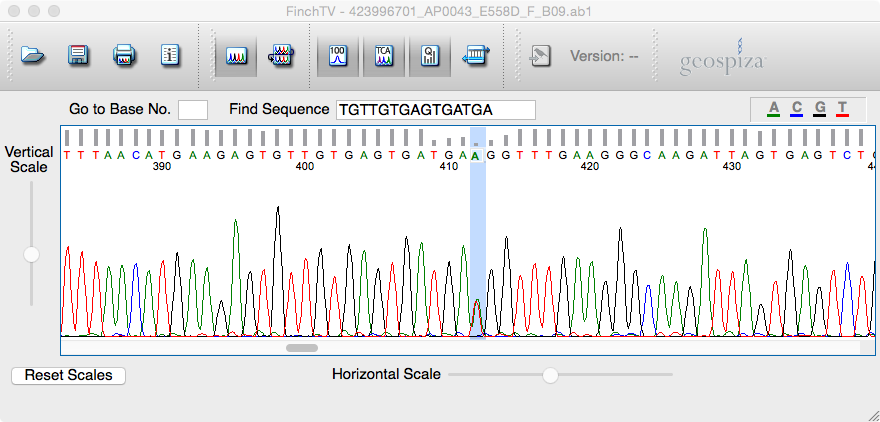

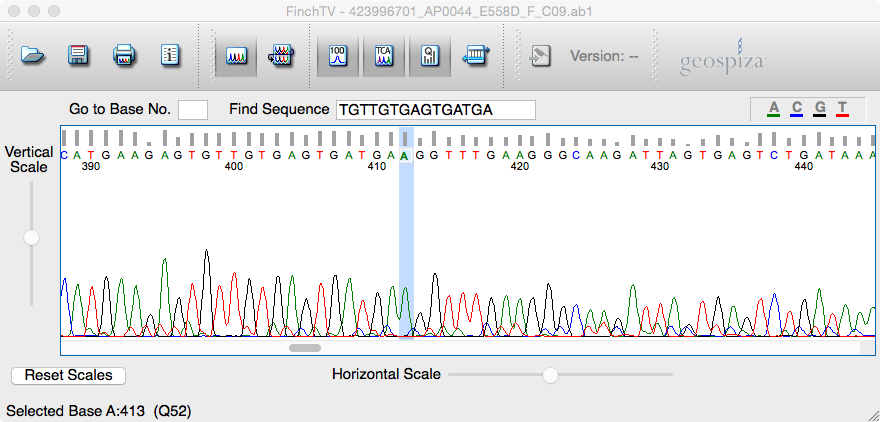


Mother

Homozygous reference

Father

Heterozygous

Proband

Heterozygous

Variant: HSPA1L:NM_005527:exon2:c.A1674T:p.Glu558Asp

Primers used: Forward ACTGCCCTGATAAAGCGCAA; Reverse GGGGCCTAGTTTTCCTGAGTC

Heterozygous status in proband and father (unaffected)

**Figure S4B. Sanger trace for Pedigree 142**


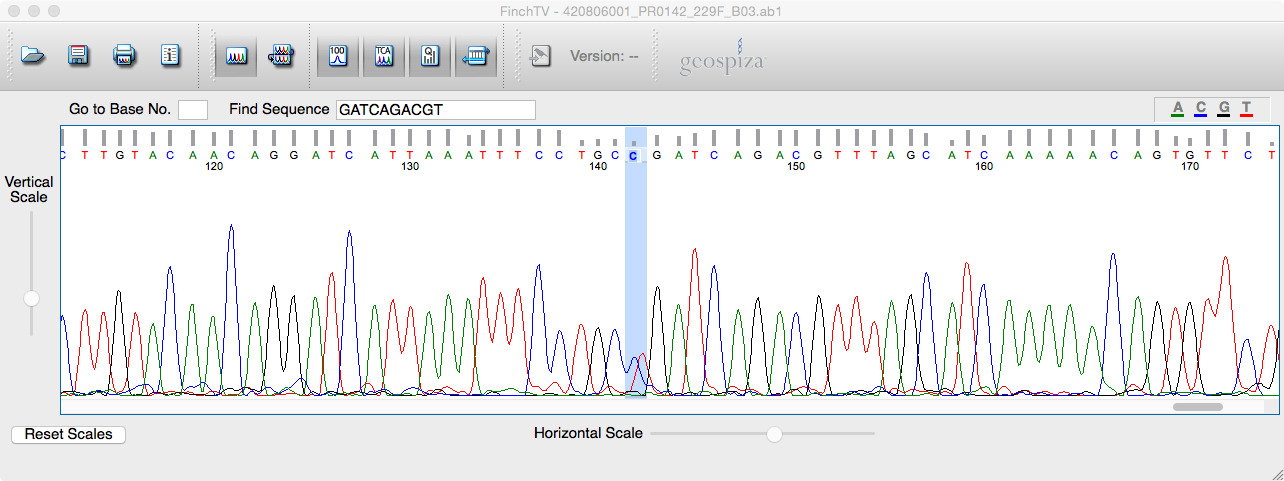

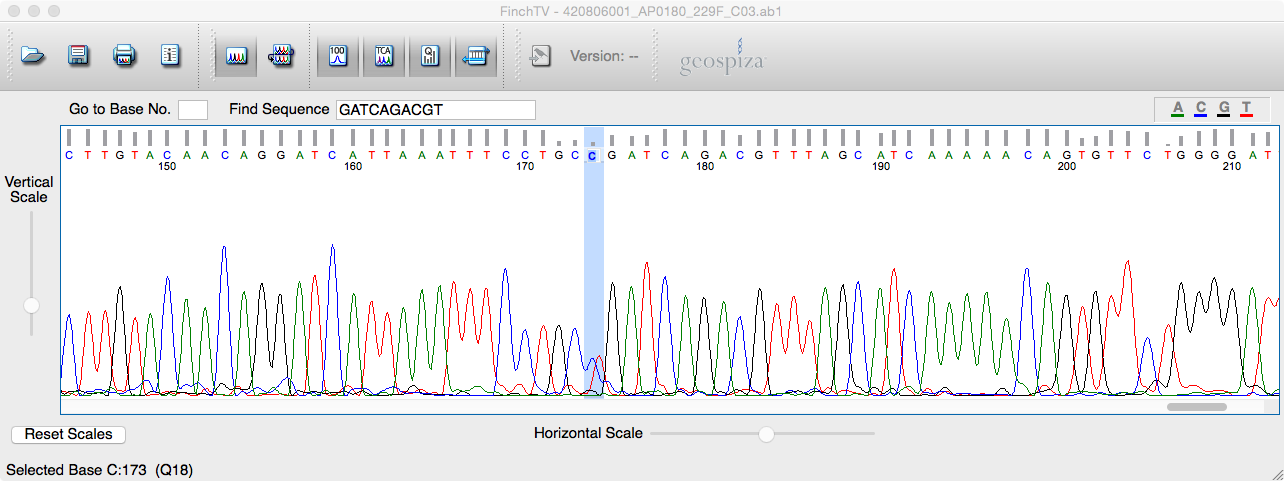

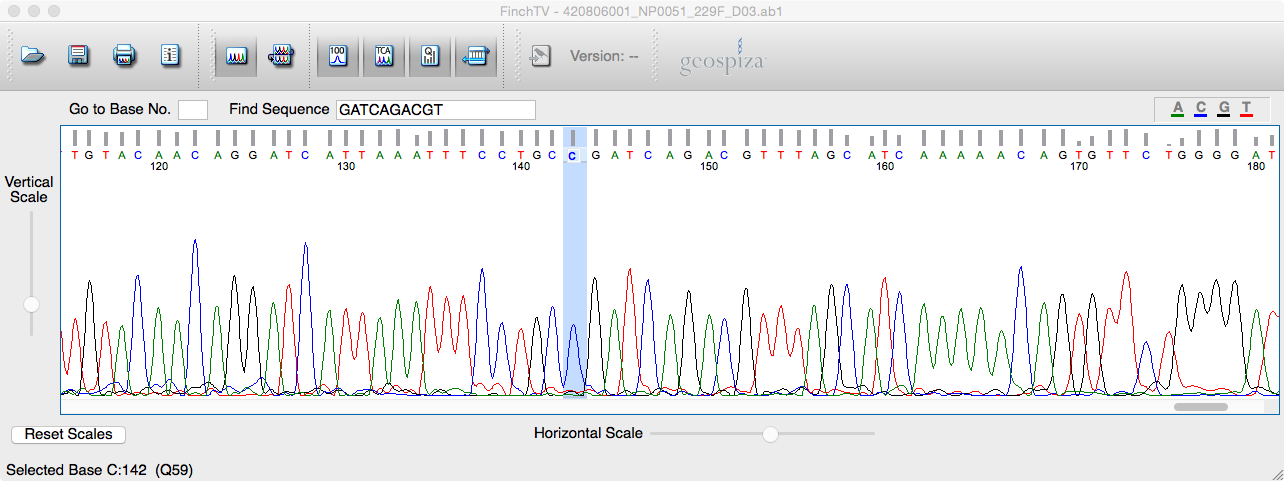


Mother

Homozygous reference

Father

Heterozygous

Proband

Heterozygous

Variant: HSPA1L:NM_005527:exon2:c.G229A:p.Gly77Ser

Primers used: Forward TTGACAACAGGCTTGTGAGC; Reverse AAATCGAGCTCTGGTGATGG

Heterozygous status in proband and father (unaffected)

**Figure S4C. Sanger trace for Pedigree 151**


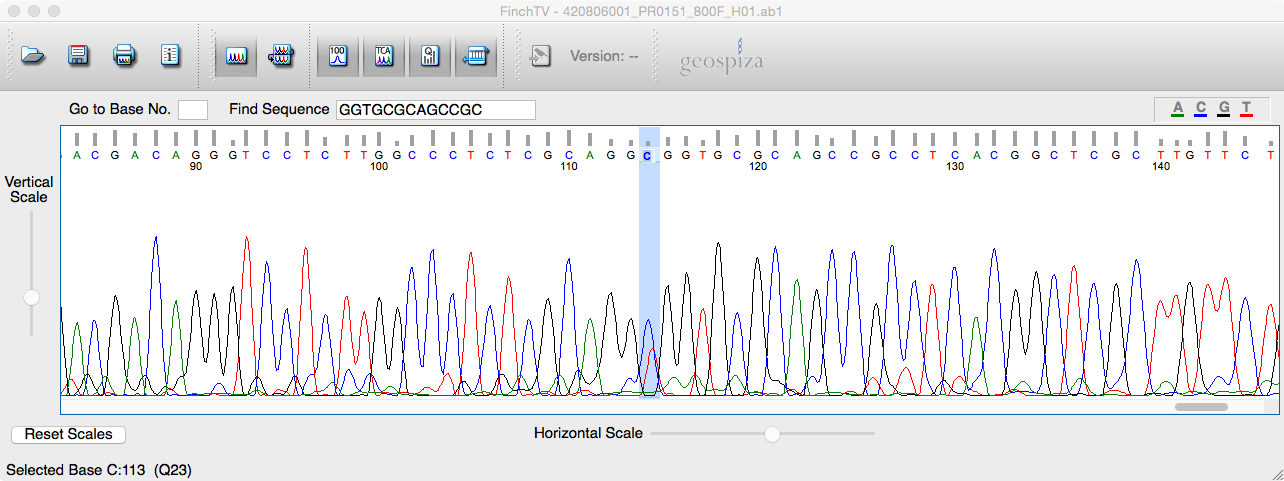

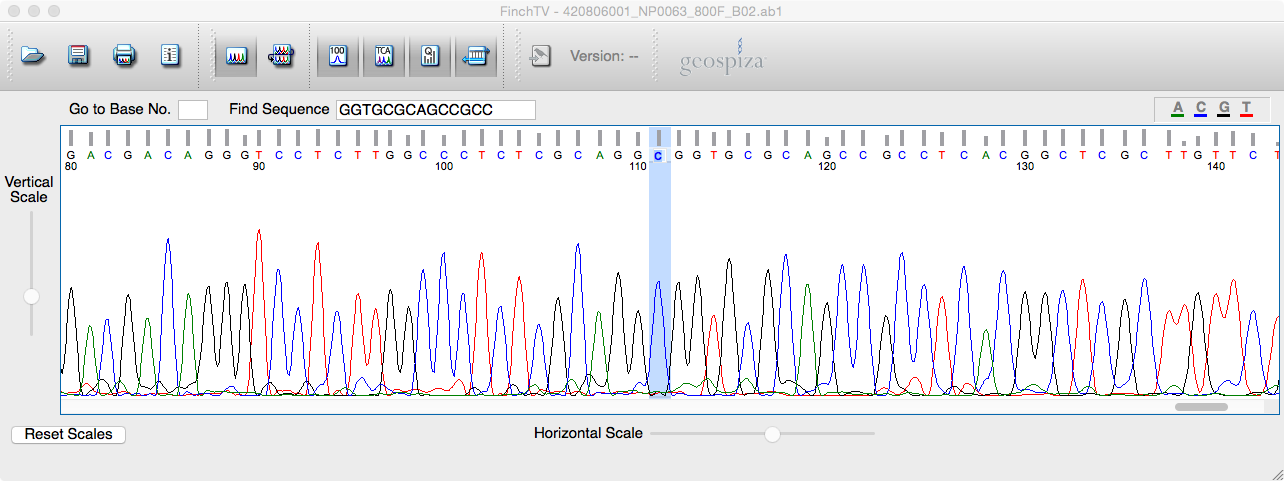

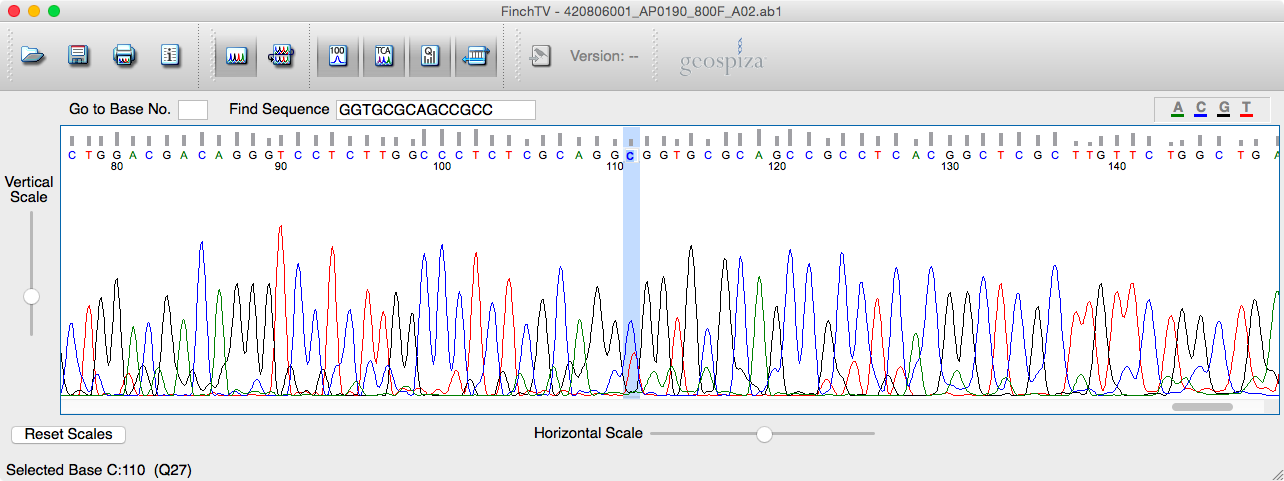


Proband

Heterozygous

Father

Homozygous reference

Mother

Heterozygous

Variant :HSPA1L:NM_005527:exon2:c.G802A:p.Ala268Thr

Primers used: Forward TTGACAACAGGCTTGTGAGC; Reverse AAATCGAGCTCTGGTGATGG

Heterozygous status in proband and mother (unaffected)

**Figure S4D. Sanger trace for Pedigree 156**


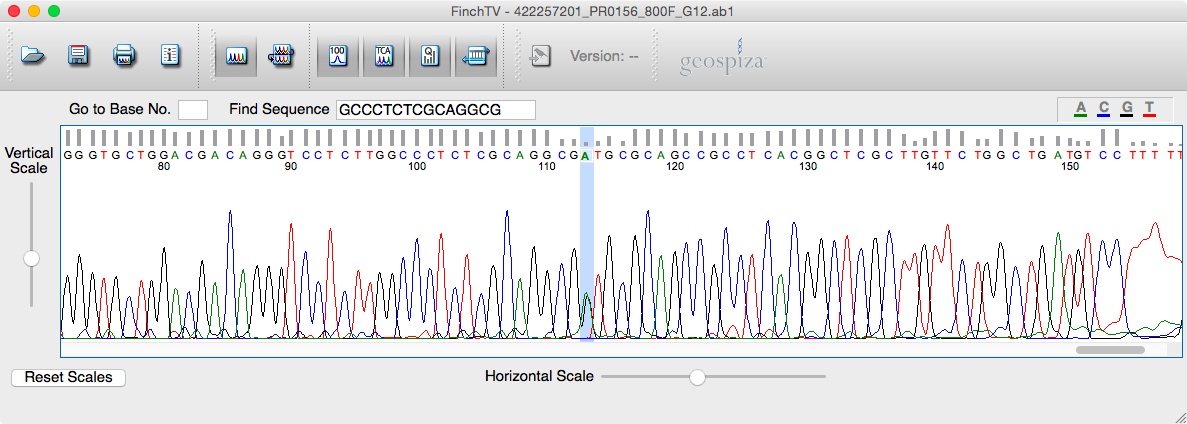


Proband

Heterozygous


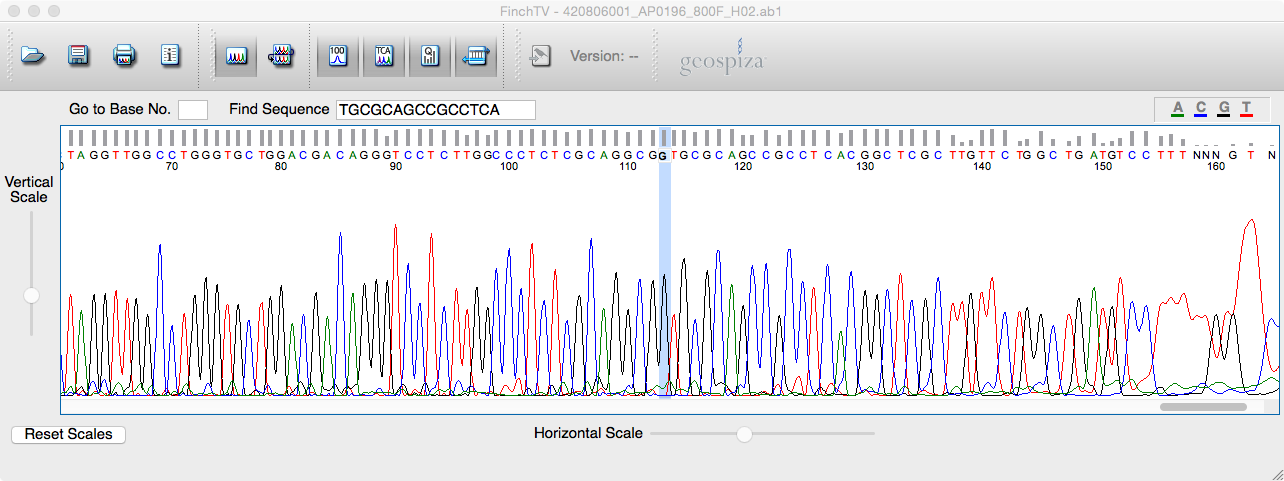

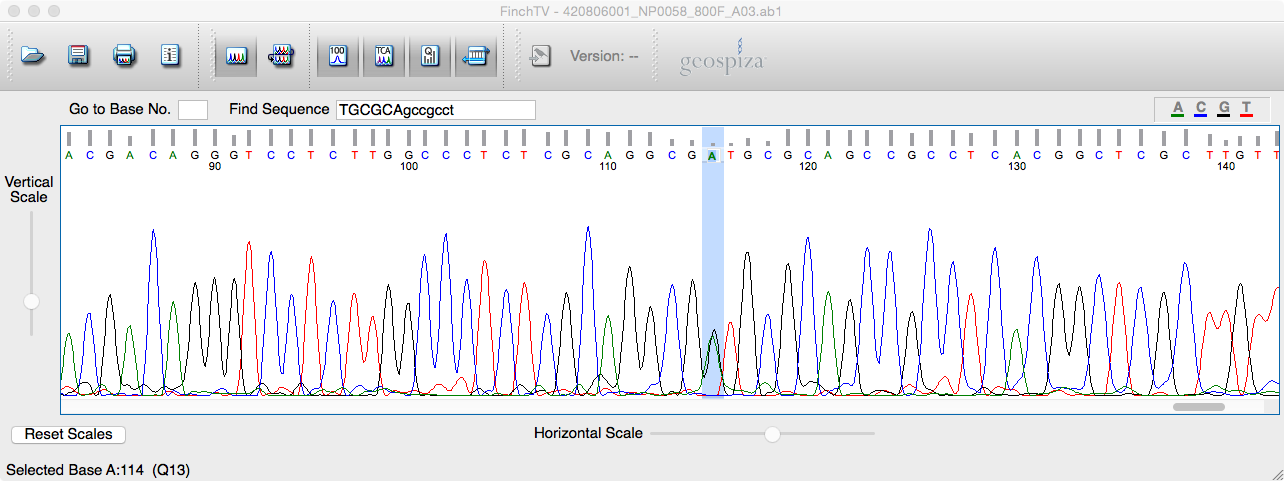


Mother

Heterozygous

Father

Homozygous reference

Variant: HSPA1L:NM_005527:exon2:c.C800T:p.Thr267Ile

Primer: Forward TTGACAACAGGCTTGTGAGC; Reverse AAATCGAGCTCTGGTGATGG

Heterozygous status in proband and mother (unaffected)

**Figure S4E. Sanger trace for Pedigree 161**


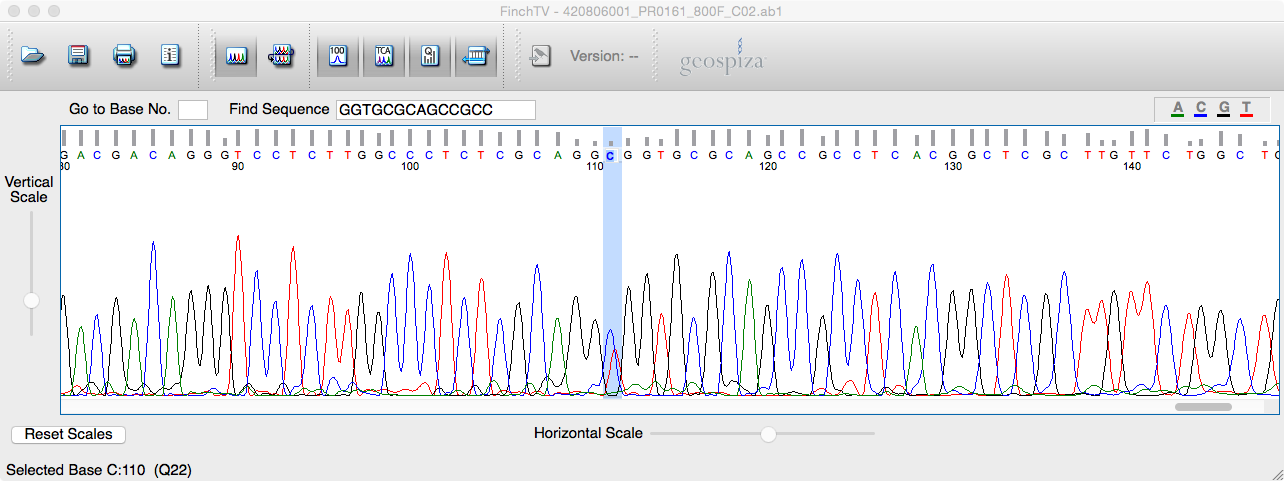


Proband

Heterozygous


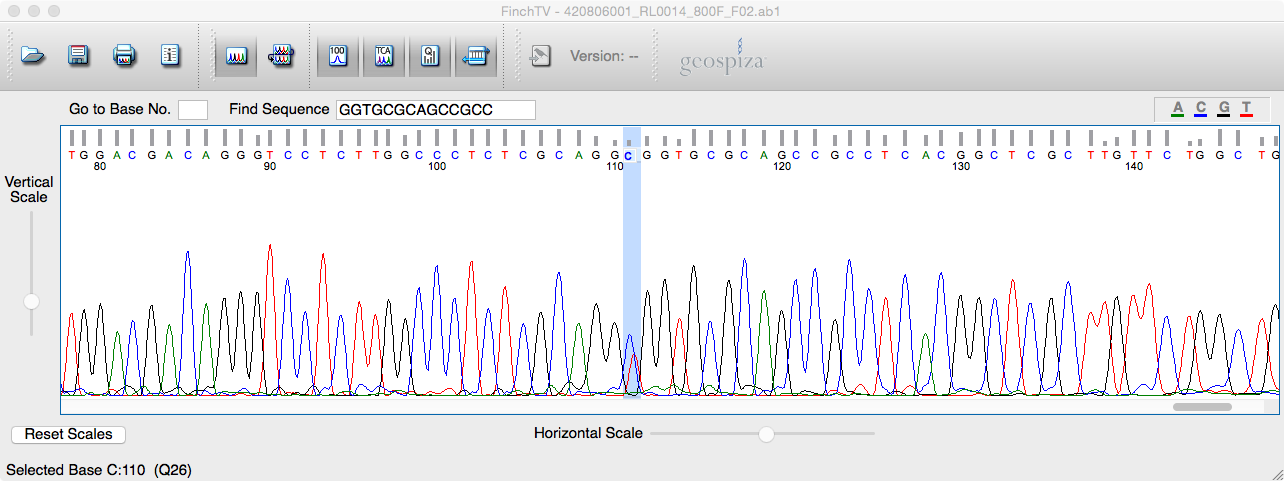

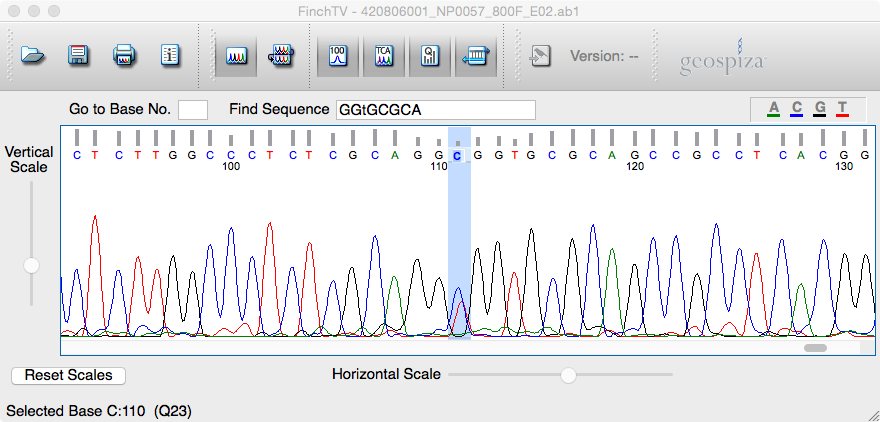

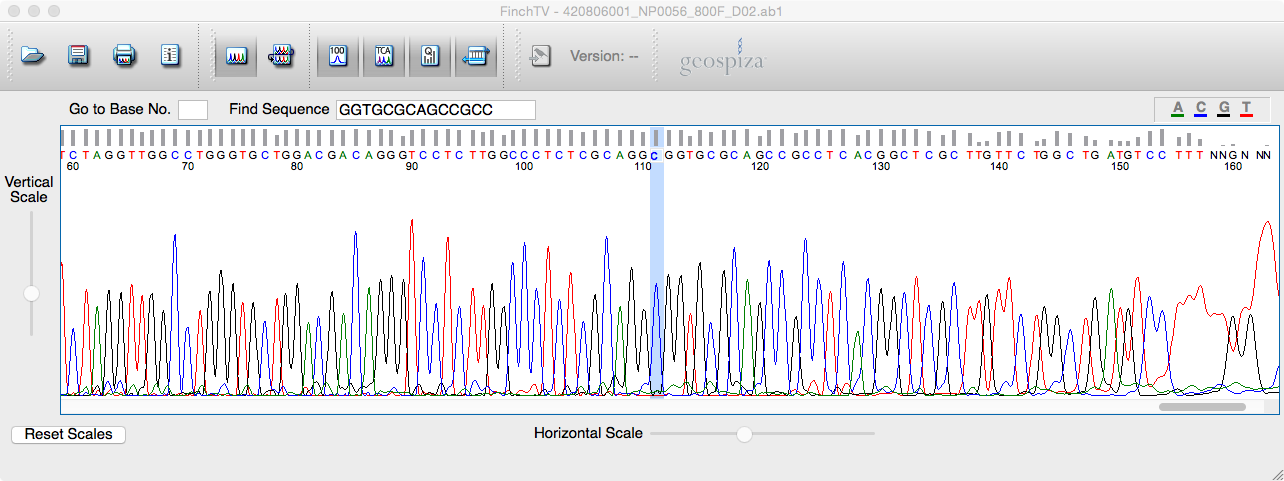


Mother

Homozygous reference

Father

Heterozygous

Sister

Heterozygous

Variant : HSPA1L:NM_005527:exon2:c.G802A:p.Ala268Thr

Primers used: Forward TTGACAACAGGCTTGTGAGC; Reverse AAATCGAGCTCTGGTGATGG

Heterozygous status in proband, unaffected father (unaffected) and sister (ulcerative colitis)

**S4F. Sanger trace for Pedigree 244**


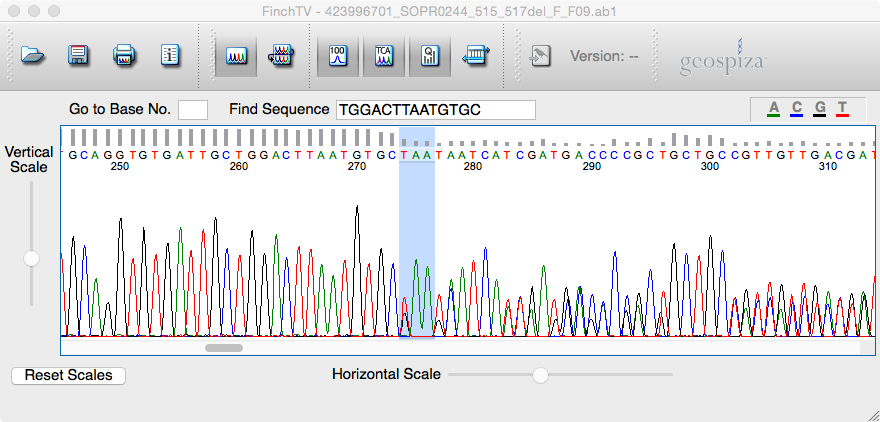

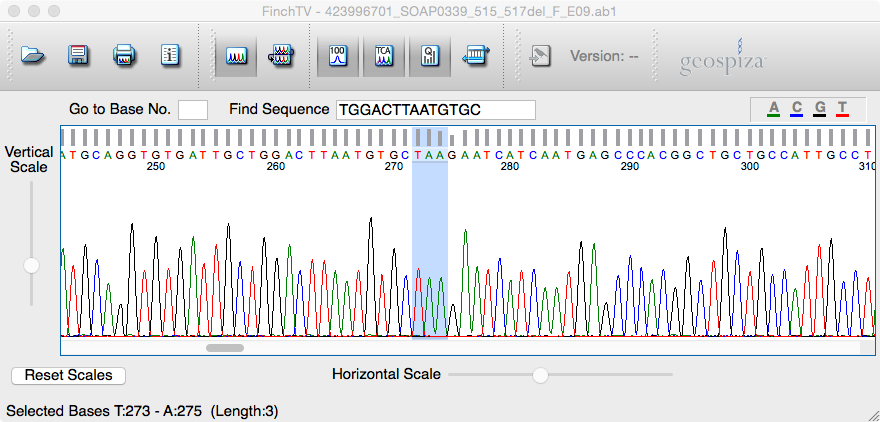


Proband

Heterozygous

Mother

Homozygous reference

Variant: HSPA1L:NM_005527:exon2:c.515_517del:p.Leu172del

Primers used: Forward ACTGCCCTGATAAAGCGCAA; Reverse GGGGCCTAGTTTTCCTGAGTC

Heterozygous status in proband

**Figure S4G. Pedigree Family A**

**Sanger trace for Family A is shown in Fig. 1A**

Primers used: Forward TAGATGATGGGATTTTTGAGGTA; Reverse CTACTAAAACAATGTCATGGATTTT
