# Supplementary material for: De novo and rare mutations in the HSPA1L heat shock gene associated with inflammatory bowel disease
Source: Genome Med. 2017 Jan 26;9:8. doi: 10.1186/s13073-016-0394-9 (PMC5270254; doi:10.1186/s13073-016-0394-9)
Supplement: Additional file 5: — Multidimensional scaling (MDS) across five ethnic groups from 1000 Genome Project, 146 pediatric IBD cases, and 126 non-IBD controls. (DOCX 261 kb) [file 13073_2016_394_MOESM5_ESM.docx]

Additional file 5. Multi-dimensional scaling (MDS) across five ethnic groups from 1000 genome project, 146 paediatric IBD cases and 126 non-IBD controls.

**
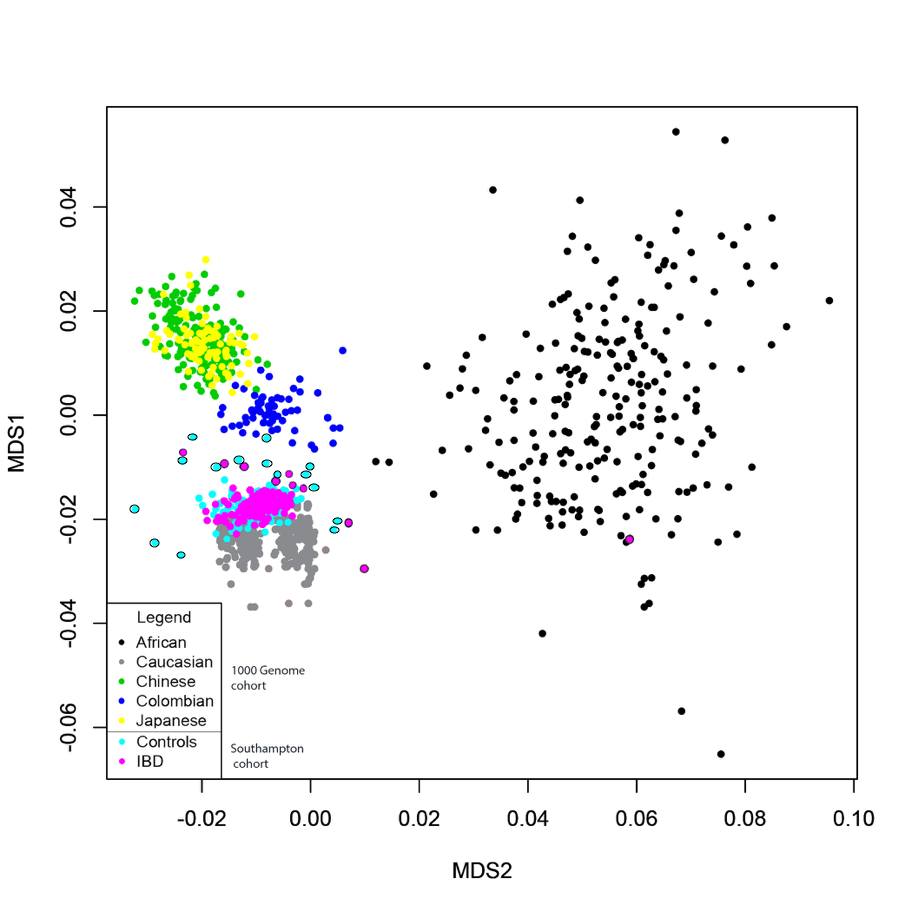
**

The five ethnic groups from 1000 Genome are colored as indicated. The Southampton IBD cohort and controls are in pink and light blue respectively. Southampton IBD samples excluded from the SKAT-O test because of ethnic status are represented with a black outline.
