# Supplementary material for: De novo and rare mutations in the HSPA1L heat shock gene associated with inflammatory bowel disease
Source: Genome Med. 2017 Jan 26;9:8. doi: 10.1186/s13073-016-0394-9 (PMC5270254; doi:10.1186/s13073-016-0394-9)
Supplement: Additional file 6: — Homozygous and heterozygous mutations unique to the index patient with ulcerative colitis (12 s). (DOCX 126 kb) [file 13073_2016_394_MOESM6_ESM.docx]

**Additional file 6.** Homozygous and heterozygous mutations unique to the index patient with ulcerative colitis (12s)

| Band | 12f | 12m | 12b | 12s | Gene | AAChange | esp6500_all | 1000g2012apr_all | snp138 | SIFT_score | PolyPhen2_HDIV_score | note |
| --- | --- | --- | --- | --- | --- | --- | --- | --- | --- | --- | --- | --- |
| 3q29 | 0/1 | 0/2 | 0/1 | 1/2 | MUC4 | p.Thr181Ile | NA | NA | rs729593 | 0.03 | 0.311 | Mucin 4, Cell Surface Associated |
| 8p23.1 | 0/1 | 0/1 | 0/1 | 1/1 | RP1L1 | p.Ala1319Gly | NA | NA | rs4840501 | 1 | 0 | Retinitis Pigmentosa 1-Like 1 |
| 11p11.2 | 0/2 | 0/1 | 0/2 | 1/2 | OR4B1 | p.Thr274Met | NA | NA | rs7130086 | 0 | 1 | Olfactory Receptor |
| 11p15.4 | 0/1 | 0/1 | 0/1 | 1/1 | OR51I2 | p.Arg263Cys | 0.020006 | 0.01 | rs75620804 | 0.01 | 1 | Olfactory Receptor |
| 11q25 | 0/1 | 0/1 | 0/1 | 1/1 | NCAPD3 | p.Arg622Gln | 0.04209 | 0.03 | rs12292394 | 0.59 | 0 | Condensin-2 complex subunit D3 |
| 14q31.1 | 0/1 | 0/1 | 0/1 | 1/1 | TSHR | p.Pro52Thr | 0.046594 | 0.03 | rs2234919 | 0.5 | 0.007 | Thyroid Stimulating Hormone Receptor |
| 22q13.2 | 0/1 | 0/1 | 0/0 | 1/1 | EFCAB6 | p.Thr1030Pro | 0.041827 | 0.03 | rs34955597 | 0.27 | 0.168 | EF-hand calcium binding domain 6 |
| 1p36.23 | 0/0 | 0/0 | 0/0 | 0/1 | SLC45A1 | p.Ala565Val | NA | NA | NA | 0.18 | 0.607 | Solute Carrier Family 45, Member 1 |
| 2p14 | 0/0 | 0/0 | 0/0 | 0/1 | SLC1A4 | p.Pro22Leu | 0.000786 | NA | rs201175768 | 0.29 | 0.1 | Transporter for Ala, Ser, Cys, and Thr |
| 5q14.1 | 0/0 | 0/0 | 0/0 | 0/1 | MSH3 | p.Pro63Ala | NA | NA | rs2405876 | . | 0.235 | Post-replicative DNA mismatch repair system |
| 5q14.1 | 0/0 | 0/0 | 0/0 | 0/1 | MSH3 | p.Pro64Ala | NA | NA | rs2405877 | . | 0.043 | Post-replicative DNA mismatch repair system |
| 6p21.33 | 0/0 | 0/0 | 0/0 | 0/1 | MUC21 | p.Glu304Gly | NA | NA | rs201896109 | . | 0.011 | 6p21.33, Mucin 21, Cell Surface Associated |
| 6p21.33 | 0/0 | 0/0 | 0/0 | 0/1 | HSPA1L | p.Ser277Leu | NA | NA | NA | 0 | 1 | 6p21.33, HSP70-Hom |
| 7q22.1 | 0/0 | 0/0 | 0/0 | 0/1 | MUC17 | p.Pro2716Ala | NA | NA | rs34924040 | 0.92 | 0.103 | Mucin 17, Cell Surface Associated |
| 8q12.1 | 0/0 | 0/0 | 0/0 | 0/1 | RPS20 | p.Thr23Pro | NA | NA | NA | 0.03 | 0.289 | Ribosomal Protein S20 |
| 11p15.5 | 0/0 | 0/0 | 0/0 | 0/1 | MUC6 | p.Ser1842Pro | NA | NA | rs111373859 | 0.36 | 0.04 | Gastric Mucin-6 |
| 11p15.5 | 0/0 | 0/0 | 0/0 | 0/1 | MUC6 | p.Asn1686Ser | NA | NA | rs200243990 | 1 | 0 | Gastric Mucin-6 |
| 11q12.1 | 0/0 | 0/0 | 0/0 | 0/1 | OR8U1 OR8U8 | p.Gly242Ser | NA | NA | rs77614949 | 0.45 | 0.025 | Olfactory Receptor |

0/0 indicates reference homozygote; 0/1 indicates heterozygote (reference/alternative); 1/1 indicates alternative homozygote; 0/2 indicates heterozygote (reference/2nd alternative); 1/2 indicates heterozygote (alternative/2nd alternative). esp6500_all, alternative allele frequency in all subjects in the NHLBI-ESP project with 6500 exomes; 1000g2012apr_all, alternative allele frequency data in 1000 Genomes Project
