# Supplementary material for: De novo and rare mutations in the HSPA1L heat shock gene associated with inflammatory bowel disease
Source: Genome Med. 2017 Jan 26;9:8. doi: 10.1186/s13073-016-0394-9 (PMC5270254; doi:10.1186/s13073-016-0394-9)
Supplement: Additional file 7: — HSPA1A and HSPA1B variants identified in patients wth IBD and controls (no filtering applied). (DOCX 68 kb) [file 13073_2016_394_MOESM7_ESM.docx]

Additional file 7. *HSPA1A* and *HSPA1B* variants identified in IBD patients and controls (no filtering applied)

| Gene | chr | Base pair location in hg19 | Var | Nucleotide change | Protein change | dbSNP137 | Frequency in 1KG Project | Cases* genotypes (homozygous reference allele, heterozygous, homozygous alternative allele)* | Controls+ genotypes (homozygous reference allele, heterozygous, homozygous alternative allele) | MAF within cases and controls |
| --- | --- | --- | --- | --- | --- | --- | --- | --- | --- | --- |
| HSPA1A | 6 | 31783755 | sn | c.222T>C | p.Ile74Ile | rs1043620 | 0.95 | 125,0,11 | 95,1,10 | 0.08884 |
| HSPA1A | 6 | 31785228 | sn | c.1695G>C | p.Ala565Ala | rs33998554 | 0.83 | 27,32,77 | 10,30,66 | 0.28099 |
| HSPA1B | 6 | 31795745 | sn | c.18G>A | p.Ala6Ala | rs34004874 | 0.01 | 134,2,0 | 104,1,1 | 0.01033 |
| HSPA1B | 6 | 31795949 | sn | c.222T>C | p.Ile74Ile | rs140434649 | 1 | 121,0,15 | 93,0,13 | 0.11570 |
| HSPA1B | 6 | 31797272 | sn | c.1545C>A | p.Ile515Ile | rs17854926 | 0.04 | 135,1,0 | 106,0,0 | 0.00206 |
| HSPA1B | 6 | 31797422 | sn | c.1695G>C | p.Ala565Ala | rs33998554 | 0.01 | 134,2,0 | 103,3,0 | 0.01033 |
| HSPA1B | 6 | 31797587 | sn | c.1860C>G | p.Gly620Gly | rs539689 | 0.56 | 34,62,40 | 36,47,23 | 0.48554 |

*****Occurrence in Soton PIBD exomes (n = 136)

**+**Occurrence in Soton control exomes (n = 106)

sn, synonymous
