# Supplementary material for: De novo and rare mutations in the HSPA1L heat shock gene associated with inflammatory bowel disease
Source: Genome Med. 2017 Jan 26;9:8. doi: 10.1186/s13073-016-0394-9 (PMC5270254; doi:10.1186/s13073-016-0394-9)
Supplement: Additional file 8: — Results of SKAT-O within HSPA1L using non-synonymous and non-frameshift rare (MAF < 0.01) variants. (DOCX 49 kb) [file 13073_2016_394_MOESM8_ESM.docx]

**Additional file 8: Results of SKAT-O within HSPA1L using non-synonymous and non-frameshift rare (MAF < 0.01) variants.**

| Gene | bp position (hg19) | Total number of samples (136 cases; 106 controls) | Frequency of individuals with rare (MAF < 0.01)* variants | Number of all variants defined in the group file | Number of variants defined as rare (MAF < 0.01)* | *P* value unadjusted |
| --- | --- | --- | --- | --- | --- | --- |
| HSPA1L | 6:31778076-31779521 | 242 | 0.024793 | 5 | 5 | 0.02428 |

* These variants received different weights in the SKAT-O joint test. The minor allele frequency is calculated within the SKAT-O software and is therefore based on the actual sample of cases and controls
