# Supplementary material for: De novo and rare mutations in the HSPA1L heat shock gene associated with inflammatory bowel disease
Source: Genome Med. 2017 Jan 26;9:8. doi: 10.1186/s13073-016-0394-9 (PMC5270254; doi:10.1186/s13073-016-0394-9)
Supplement: Additional file 9: — Results of SKAT-O within HSPA1L using non-synonymous and non-frameshift variants, excluding common variants (MAF > 0.05). (DOCX 51 kb) [file 13073_2016_394_MOESM9_ESM.docx]

Additional file 9. Results of SKAT-O within *HSPA1L* using non-synonymous and non-frameshift variants, excluding common variants (MAF_1KG_ >0.05).

| Gene | bp position (hg19) | Total number of samples (136 cases; 106 controls) | Frequency of individuals with rare (MAF < 0.01)* variants | Number of all variants defined in the group file | Number of variants defined as rare (MAF < 0.01)* | P value unadjusted |
| --- | --- | --- | --- | --- | --- | --- |
| HSPA1L | 6:31778076-31779728 | 242 | 0.033058 | 7 | 6 | 0.034846 |

* These variants received different weights in the SKAT-O joint test. The minor allele frequency is calculated within the SKAT-O software and is therefore based on the actual sample of cases and controls.
